# Satisfactory results of a psychometric analysis and calculation of minimal clinically important differences of the World Health Organization quality of life-BREF questionnaire in an observational cohort study with lung cancer and mesothelioma patients

**DOI:** 10.1186/s12885-018-4793-8

**Published:** 2018-11-26

**Authors:** Mark de Mol, Sabine Visser, Joachim G. J. V. Aerts, Paul Lodder, Jolanda de Vries, Brenda L. den Oudsten

**Affiliations:** 1 Department of Pulmonary Diseases, Amphia Hospital, Molengracht 21, 4818 CK Breda, The Netherlands; 2 Department of Pulmonary Diseases, Erasmus MC Cancer Institute, Dr. Molewaterplein 50, 3015 GD Rotterdam, The Netherlands; 3 Department of Epidemiology, Erasmus MC – University Medical Centre Rotterdam, P.O. Box 2040, 3000 CA Rotterdam, The Netherlands; 4 Department of Methodology and Statistics, Tilburg University, P.O. Box 90151, 5000 LE Tilburg, The Netherlands; 5 Department of Medical and Clinical Psychology, Centre of Research on Psychological and Somatic Disorders (CoRPS), Tilburg University, P.O. Box 90151, 5000 LE Tilburg, The Netherlands; 6 Departement of Medical Psychology, Elisabeth-TweeSteden Hospital, Hilvarenbeekseweg 60, 5022 GC Tilburg, The Netherlands

**Keywords:** World Health Organization quality of life-BREF, Lung cancer, Quality of life, Validity, Reliability, Minimal clinically important difference

## Abstract

**Background:**

To determine the psychometric properties and minimal clinically important differences (MCIDs) of the World Health Organization Quality of Life-BREF (WHOQOL-BREF) in advanced stage lung cancer patients.

**Methods:**

Patients (*n* = 153) completed the WHOQOL-BREF and the European Organisation for Research and Treatment of Cancer Quality of Life Questionnaire Core 30 (EORTC QLQ-C30). Confirmatory factor analysis (CFA) was performed and reliability and construct validity determined. MCIDs were estimated with two distribution-based methods (0.5 standard deviation (SD) and 1 standard error of measurement (1 SEM)).

**Results:**

CFA confirmed WHOQOL-BREF domain structure. All domains demonstrated good internal consistency (α > 0.70), except Social Relationships (α = 0.57). Nineteen of the 24 WHOQOL-BREF items had correlations of ≥ 0.40 with their intended domain. Four items had higher correlations with a domain other than their intended domain. Moderate to strong correlations were observed for corresponding domains of the two questionnaires, except for the social domains (*r* = 0.07). For 0.5 SD, MCIDs ranged from 0.88 to 1.55, and for 1 SEM MCIDs ranged from 1.76 to 2.72.

**Conclusions:**

The WHOQOL-BREF has satisfactory psychometric properties in patients with advanced stage lung cancer, whereas the observed MCIDs provide a method for interpretation of scores.

## Background

In general, chemotherapy in patients with advanced disease lung cancer is associated with small survival benefits [1, 2]. In addition, Quality of Life (QoL) may be reduced in patients with lung cancer [3]. This emphasizes the importance of maintaining patients’ Quality of Life (QoL) at an acceptable level by early identification of treatment-induced changes. QoL is evaluated by questionnaires of which the European Organisation for Research and Treatment of Cancer Quality of Life-Core 30 questionnaire (EORTC QLQ-C30) is one of the most frequently applied in cancer [4]. However, this instrument is considered to be a Health Related Quality of Life (HRQoL) questionnaire (i.e. it focusses on those aspects of QoL related to the disease and its treatment) and measures to a lesser extent patients’ opinions of the other aspects of QoL [4]. Therefore, the WHO formulated a comprehensive definition of QoL based on extensive research. In 2004, they released the World Health Organization Quality of Life instrument-BREF (WHOQOL-BREF) to enable rapid QoL assessment in epidemiological surveys and clinical studies [5].

Recently, a study performed in Taiwanese patients diagnosed with stage I to IV lung cancer reported satisfactory psychometric properties of the WHOQOL-BREF. However, the 28-item Taiwanese version of the WHOQOL-BREF (the original WHOQOL-BREF holds 26 items [5]) was used and specific results concerning patients with advanced disease lung cancer were not reported. Therefore, further psychometric validation of the WHOQOL-BREF in this group may be mandatory for several reasons. First, patients with advanced lung cancer form a well-defined group due to their poor prognosis compared to patients with stage I or II lung cancer and the population in the WHOQOL-BREF field trial [5]. Second, apart from the symptoms of lung cancer, treatment is in most patients with advanced disease lung cancer associated with substantial adverse events which can directly influence (HR) QoL. Third, although some studies have reported results of the WHOQOL-BREF in lung cancer [6, 7], the application of this questionnaire in patients starting treatment with chemotherapy was not reported. Fourth, as correct interpretation of the minimal clinically important difference (MCID) depends on the psychometric characteristics of the instrument and the patient population from which it is derived, the determination of a reliable MCID in lung cancer ideally requires evaluation of the reliability and validity of the WHOQOL-BREF in these patients.

Given these considerations***,*** additional research is needed to enable implementation of the WHOQOL-BREF in future trials investigating therapeutic regimens in lung cancer and to facilitate the interpretation of individual scores. To contribute to these goals the objective of our study focused on two main aspects of the WHOQOL-BREF: (1) to test the reliability and validity of the WHOQOL-BREF in patients with advanced disease lung cancer, and (2) to assess the MCIDs of the WHOQOL-BREF domain scores.

We expected that the 4-domain structure of the WHOQOL-BREF would be confirmed and that the internal consistencies of all domains were at least acceptable, except for Social Relationships [5]. Moreover, we hypothesized that all items of the WHOQOL-BREF would have an acceptable positive correlation (i.e. correlation coefficient ≥ 0.40) with their intended domains and that all items would have higher positive correlations with their intended domain than with the other three domains [8]. Considering construct validity, we expected significant differences in mean domain scores between known groups according to ECOG performance score and EORTC QLQ-C30 Global Health Status/QoL score [9]. In addition, construct validity was assessed by correlating the domains of the WHOQOL-BREF with the scales of the EORTC QLQ-C30 [9]. We hypothesized that all domains would have at least moderate correlations (i.e. correlation coefficient ≥ 0.50) with their corresponding scales of the EORTC QLQ-C30 [10]. In this study, we expected no floor or ceiling effects for domain scores of the WHOQOL-BREF.

## Methods

### Study population

PERSONAL is a prospective observational multi-center cohort study of patients with non-squamous non-small cell lung carcinoma (NSCLC) and unresectable mesothelioma receiving pemetrexed. Patients were recruited from October 2012 to November 2014 from three teaching hospitals (Erasmus University Medical Center, Amphia Hospital and Sint Franciscus Gasthuis hospital) and a regional hospital (Bravis hospital) in the Netherlands. For this study, which is part of an ongoing analysis of PERSONAL, data of 191 enrolled patients was available. Patients were enrolled if they met the following criteria: were aged 18 years or older, had a cytological or histological confirmed diagnosis of non-squamous NSCLC or unresectable malignant pleural mesothelioma and started treatment with pemetrexed monotherapy or in combination with a platinum compound. Patients were excluded when they were not able to read Dutch or could not complete the questionnaires because of a physical or mental condition. A sample size of at least 50 patients was needed in order to perform a validation study [9]. Informed consent was obtained from all individual participants included in the study. This multi-center study was approved by the Institutional Review Board of the Erasmus University Medical Center in Rotterdam, the Netherlands.

### Study measures

The WHOQOL-BREF [5, 11] is a well-established generic QoL instrument intended for use in a wide range of chronic diseases and cancer [5]. It comprises 24 items divided over four domains plus two items of the General Facet describing Overall QoL and General Health. The domains represent Physical Health (seven items), Psychological Health (six items), Social Relationships (three items), and Environment (eight items) and are scored on a 4–20 scale with higher scores indicating a better QoL [11]. The General Facet is scored on a 2–10 scale. Previous studies have demonstrated good psychometric properties of the WHOQOL-BREF in patients with lung cancer [12] and in patients with chronic diseases or different forms of cancer [5].

The EORTC QLQ-C30 is a cancer-specific HRQoL instrument with demonstrated psychometric properties [13]. It consists of 30 items and incorporates a Global Health Status/QoL scale, five functional scales and several single items assessing additional symptoms or problems. The functional scales represent Physical Functioning (five items), Cognitive Functioning (two items), Emotional Functioning (four items), Role Functioning (two items), and Social Functioning (two items). EORTC QLQ-C30 scales are scored on a 0–100 scale, with higher scores on the functional scales being indicative of better HRQoL, whereas higher scores on the symptom scales are reflective of worse symptoms [4].

All questionnaires were completed after diagnosis and before the first cycle of chemotherapy. In addition to completing the questionnaires, we collected sociodemographic information (i.e. age, gender, educational level, ethnicity, employment, partner status) and clinical information (i.e. cancer stage, type of tumour, line of therapy and the Eastern Cooperative Oncology Group (ECOG) performance status). At day 1 of the first cycle of chemotherapy we assessed, according to Common Terminology Criteria for Adverse Events (CTCAE) version 3.0, the severity and number of different cancer related and, if applicable, treatment related adverse events that patients experienced.

### Statistical analysis

The response distributions of item and domain scores of the WHOQOL-BREF were assessed by using two methods. As proposed in the validation paper of the WHOQOL-BREF, skewness was observed if less than 10% of responses fell in each of two adjacent scale points of an item at the extreme ends of the scale [5]. Floor and ceiling effects of domain scores of the WHOQOL-BREF were considered to be present if more than 15% of the respondents achieved the lowest (i.e. floor effect) or highest (i.e. ceiling effect) possible score [9].

The multi-trait/multi-method methodology, as proposed by Campbell and Fiske [14] and later adapted by Ware et al., was used to study item-domain relations [15]. Analyses were performed with MAP-R software which examines the correlations between items and domains and corrects the correlation of each item with its intended domain for overlap [15]. For the multi-trait/multi-item analyses, missing values are replaced by the mean score of the other items of the corresponding domain if at least half of the items are completed.

According to Trask et al., item-convergent validity was defined as a correlation coefficient ≥ 0.40 between questionnaire items and their intended domains [8]. Item-divergent validity was supported when items had higher correlations with their intended domain than with other domains of the questionnaire [8].

Construct validity was evaluated by correlating the WHOQOL-BREF domains with the corresponding scales of the EORTC QLQ-C30 using Pearson’s correlation coefficient. According to Hinkle, correlations of 0.00 to 0.30 were regarded as negligible, 0.30 to 0.50 as low, 0.50 to 0.70 as moderate, 070 to 0.90 as high, and correlations of 0.90 to 1.00 as very high [10]. In addition, known-groups validity comparisons were made for the WHOQOL-BREF domains in relation to the total number of different adverse events, the number of different grade 3 or 4 adverse events, the ECOG performance status and the Global Health Status/QoL score of the EORTC QLQ-C30 to assess construct validity. One-way ANOVA was used to determine whether there were any significant differences between the means of the groups.

**Fig. 1:**
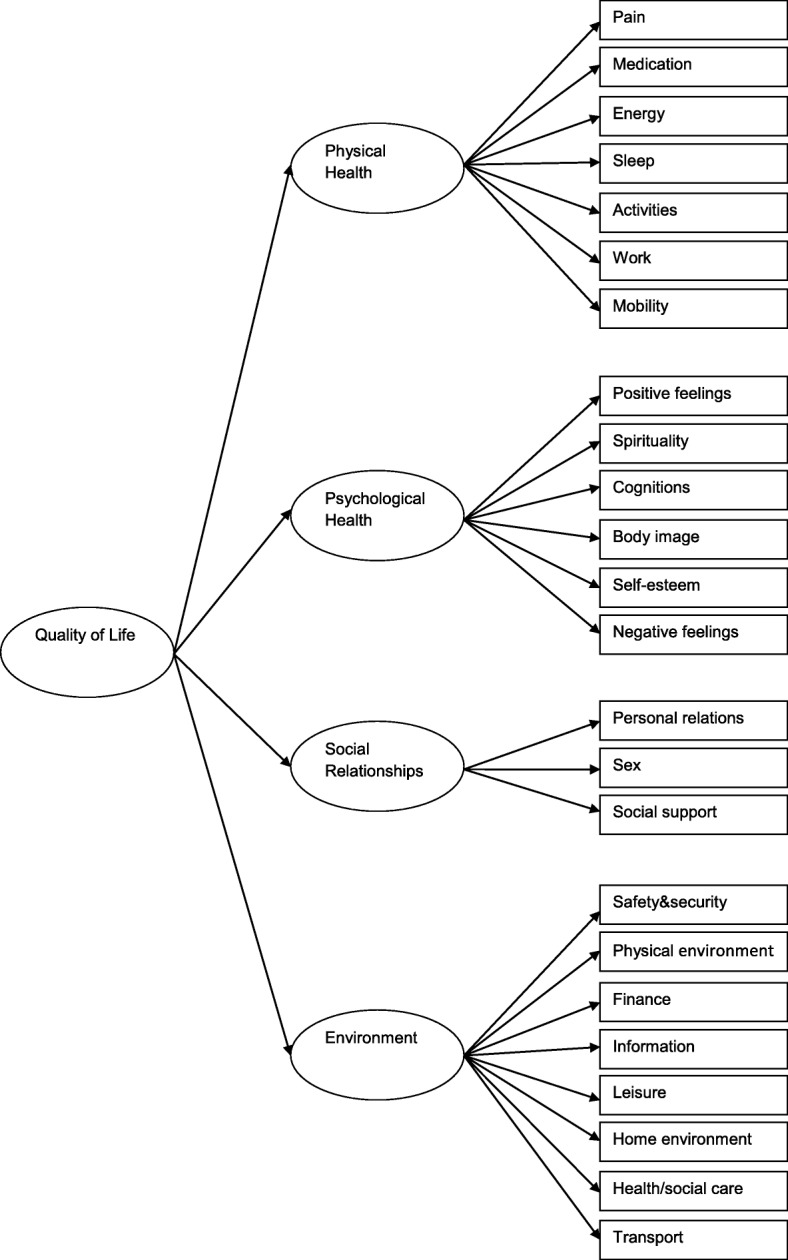
Four factor model of the WHOQOL-BREF**.** Abbreviations: WHOQOL-BREF, World Health Organization Quality of Life questionnaire

Internal consistency reflects the capability of items within a domain to measure the same concept. To evaluate internal consistency, first the four-factor design of the WHOQOL-BREF was analysed with confirmatory factor analysis (CFA) using structural equation modelling. Missing values were replaced by expectation-maximization imputation for the CFA. The original model is demonstrated in Fig. 1. Goodness of fit was assessed by the Comparative Fit Index (CFI) and the Root Mean Square Error Approximation (RMSEA). A satisfactory to good fit is defined when CFI > 0.90 and RMSEA < 0.06 [16, 17]. For the resulting domains, Cronbach’s coefficient alpha was calculated to express internal consistency. A coefficient of 0.70 or higher was considered to be acceptable [9].

For each WHOQOL-BREF domain, the MCID was calculated using two distribution-based methods (i.e., the 0.5 SD [18] and 1 standard error of measure (SEM) [19–21]). MCID is the smallest change in an outcome that a patient would identify as important. The 0.5 SD benchmark of an outcome measure means that patients improving more than 0.5 of the outcome score’s SD have reached a minimal clinically important difference [22]. As we lacked a test-retest reliability coefficient, we used the conservative lower bound of the 95% confidence intervals of the Cronbach’s alphas of the four domains to calculate the SEM. Thus, the SEM was calculated with an altered version of the SEM formula [23]: SD x √(2× (1 – lower bound 95% Confidence Interval Cronbach’s alpha)). A *p*-value of *p* < 0.05 was considered to be statistically significant. Analyses were performed using SPSS version 21.0, except for the CFA (AMOS version 22.0) and the calculation of the 95% confidence intervals of the four domains of the WHOQOL-BREF (R, version 3.2.5).

## Results

### Patient characteristics

Of the 191 enrolled patients, 153 patients (80.1%) completed the questionnaires to a sufficient degree. Table 1 summarizes the patient characteristics of these patients.

**Table 1:** Characteristics of study population

Characteristic	Overall sample (*n* = 153)
Age, years	
Mean (SD)	63.4 (9.2)
Min, max	37, 83
Sex	
Male	83 (54.2)
Ethnicity	
White/Caucasian	143 (93.5)
Asian	3 (2.0)
Negroid	2 (1.3)
Other	5 (3.3)
Education^a^	
Low	113 (73.9)
High	33 (21.6)
Unknown	7 (4.6)
Employment	
Yes	40 (26.1)
No	112 (73.2)
Unknown	1 (0.7)
Partner status	
Married/cohabiting	116 (75.8)
Unmarried partners/not cohabiting	6 (3.9)
Divorced/separated	14 (9.2)
Widowed/partner died	10 (6.5)
Single	6 (3.9)
Unknown	1 (0.7)
Cancer stage^b^	
Locally advanced (IIIB)	19 (12.4)
Metastatic (IV)	119 (77.8)
Other	14 (9.2)
Unknown	1 (0.7)
Type of tumor^b^	
Adenocarcinoma	141 (92.2)
Large cell carcinoma	4 (2.6)
Mesothelioma	7 (4.6)
Large cell neuroendocrine carcinoma	1 (0.7)
Line of therapy	
First line	134 (87.6)
Second line	10 (6.5)
Third line	1 (0.7)
Adjuvant	8 (5.2)
ECOG performance status^a^	
Grade 0	39 (25.5)
Grade 1	99 (64.7)
Grade 2	11 (7.2)
Grade 3	2 (1.3)
Unknown	2 (1.3)

*Abbreviations: n* number of patients, *SD* standard deviation, *ECOG* Eastern Cooperative Oncology Group

Values are given in numbers (percentages) unless stated otherwise

^a^Low education: persons whose highest level of education is primary education, lower general education or lower vocational education. High education: persons whose highest level of education is higher general education, higher vocational education or university

^b^Measured at baseline

### Mean scores, floor and ceiling effects, and skewness

The WHOQOL-BREF domain scores are shown in Table 2. The mean General Facet score was 5.9 (1.8). Mean scores of the four domains ranged from 12.9 (SD 3.1; Physical Health) to 16.2 (SD 2.6; Social Relationships). Floor and ceiling effects of the domain scores were below the limit of 15%. Fourteen of the 26 items demonstrated skewed response distributions with responses < 10% in each of two adjacent scale points at the extreme lower end of the scale indicating that most of the information was distributed over the other scale points (Table 2). These items were Positive Feelings, Spirituality, Cognitions, Self-esteem, and Body Image for the Psychological Health domain and Personal Relations and Social Support for the Social Relationships domain. In addition, all items of the Environment domain, except Leisure, demonstrated few responses at the extreme lower end of the scale. One item, Negative Feelings exhibited responses < 10% in each of two adjacent scale points at the extreme upper end of its scale.

**Table 2:** Frequency responses for items of the WHOQOL-BREF

Items/domains	Description	*n*	Mean (SD)	Floor effect (%)	Ceiling effect (%)	Scale points^a^				
						1	2	3	4	5
General facet										
	Overall QoL	150	5.9 (1.8)	4 (2.7)	3 (2.0)	3.3	14.4	24.2	44.4	12.4
	General Health					21.6	35.9	26.1	13.1	2.6
Physical Health										
3	Pain	153	12.9 (3.1)	1 (0.7)	1 (0.7)	28.1	28.8	19.0	20.3	3.3
4	Medication					11.8	26.8	25.5	24.8	10.5
10	Energy					3.9	19.6	35.9	25.5	14.4
15	Sleep					8.5	23.5	26.1	26.1	15.0
16	Activities					7.8	34.6	24.8	26.8	5.9
17	Work					12.4	39.2	21.6	20.3	6.5
25	Mobility					4.6	11.8	13.1	35.3	35.3
Psychological Health										
5	Positive feelings	153	14.4 (2.5)	0 (0.0)	2 (1.3)	**3.9**	**7.8**	39.2	41.2	7.8
6	Spirituality					**2.6**	**5.2**	32.7	43.8	15.0
7	Cognitions					**4.6**	**9.8**	44.4	22.2	19.0
11	Body image					**0.7**	**4.6**	28.1	26.1	39.9
18	Self esteem					**0.7**	**9.8**	30.7	43.8	13.7
26	Negative feelings					14.4	32.7	43.1	**9.8**	**0.0**
Social Relationships										
19	Personal relations	153	16.2 (2.6)	0 (0.0)	20 (13.1)	**0.7**	**0.7**	**6.5**	40.5	50.3
20	Sex					5.9	13.7	36.6	24.8	16.3
21	Social support					**0.7**	**1.3**	**9.2**	34.0	54.9
Environment										
8	Safety & security	153	15.9 (2.3)	0 (0.0)	1 (0.7)	**0.7**	**3.9**	26.8	32.0	36.6
9	Physical environment					**0.7**	**2.0**	26.8	35.3	34.0
12	Finance					**2.0**	**6.5**	37.3	21.6	32.0
13	Information					**0.0**	**0.7**	36.6	37.3	24.8
14	Leisure					3.9	13.1	24.2	35.3	23.5
22	Home environment					**2.6**	**5.2**	12.4	39.9	39.9
23	Health/social care					**1.3**	**2.0**	15.7	49.0	32.0
24	Transport					**1.3**	**2.0**	**9.2**	43.8	43.8

*Abbreviations: WHOQOL-BREF* World Health Organization Quality of Life-BREF questionnaire, *n* number of patients, *SD* standard deviation, *QoL* quality of life

^a^Values are given in percentages

Values in bold represent skewed distributions of the frequency of responses of patients

### Confirmatory factor analysis

CFA with the use of structural equation modelling was conducted to analyse the four-factor structure of the WHOQOL-BREF. Inspection of the modification indices revealed two possible modifications to improve the model fit of the original model. After adding error covariances between the measurement error of the items 1 (Pain) and 2 (Medication) and between 8 (Positive Feelings) and 9 (Spirituality) model fit improved. The CFI increased from 0.854 to 0.896 whereas the RMSEA decreased from 0.069 to 0.058 approaching both of the criteria for a satisfactory to good fit (CFI > 0.90 and RMSEA < 0.06).

### Multi-trait/multi-item analyses and internal consistency

Multi-trait/multi-item analyses demonstrated that all items, except those representing Sleep, Body Image, Sex, Physical Environment, and Finance had a correlation of ≥ 0.40 with their intended domain (Table 3). Four items showed higher correlations with other domains than their own. The item Sleep of the Physical Health domain had a higher positive correlation with the Psychological Health domain, whereas the items Body Image and Self-esteem of the Psychological Health domain had higher positive correlations with the Environment domain and the Physical Health domain respectively. In addition, the item Personal Relationships of the Social Relationships domain showed a higher positive correlation with the Environment domain than its intended domain. For all domains, except the Social Relationships domain, Cronbach’s alpha was higher than 0.70 (i.e. Physical Health: 0.81, 95% CI 0.76–0.85; Psychological Health: 0.77, 95% CI 0.71–0.83; Environment: 0.77, 95% CI 0.70–0.82; Social Relationships: 0.57, 95% CI 0.43–0.68).

**Table 3:** Multi-trait/Multi-item item-domain correlation for the WHOQOL-BREF (*n* = 153)

Items/domains	Description	Physical Health	Psychological Health	Social Relationships	Environment
Physical Health					
3R	Pain	.53^a^	.41	**.20**	**.35**
4R	Medication	.40^a^	**.24**	**.01**	**.21**
10	Energy	.57^a^	.53	**.13**	.42
15	Sleep	.27^a^	.32	**.06**	.27
16	Activities	.72^a^	.56	**.17**	**.46**
17	Work	.70^a^	**.54**	**.17**	**.43**
25	Mobility	.61^a^	.47	**.24**	**.45**
Psychological Health					
5	Positive feelings	.52	.67^a^	**.33**	**.47**
6	Spirituality	**.34**	.53^a^	**.24**	**.36**
7	Cognitions	.44	.48^a^	**.20**	.48
11	Body image	.32	.34^a^	.30	.45
18	Self esteem	.60	.58^a^	**.38**	.49
26R	Negative feelings	.43	.52^a^	**.21**	.43
Social Relationships					
19	Personal relations	**.16**	.30	.43^a^	.47
20	Sex	**.15**	.31	.32^a^	.31
21	Social support	**.16**	.28	.43^a^	.42
Environment					
8	Safety & security	.46	.57	**.36**	.60^a^
9	Physical environment	**.12**	.35	.31	.38^a^
12	Finance	.23	.29	.26	.30^a^
13	Information	**.24**	**.33**	**.36**	.53^a^
14	Leisure	.41	.34	**.19**	.46^a^
22	Home environment	.46	.55	.41	.56^a^
23	Health/social care	.36	.41	.32	.41^a^
24	Transport	**.39**	.44	.43	.57^a^

*Abbreviations: SD* standard deviation, *n* number of patients, *QoL* Quality of Life, *WHOQOL-BREF* World Health Organization Quality of Life-BREF questionnaire

^a^Pearson item-scale correlations corrected for overlap

Correlations in bold represent correlations between items and domains that differ more than two standard errors from their correlations with their own domains

### Construct validity

Table 4 presents the correlations between the domains of the WHOQOL-BREF and the EORTC QLQ-C30 domains/symptom scales. In general, low correlations were observed between WHOQOL-BREF domains and EORTC QLQ-C30 domains/symptom scales. Only for Physical Health, moderate to high correlations were observed with the EORTC QLQ-C30 domains except for the correlation with Cognitive Functioning. The lowest correlations were found between Social Relationships and the EORTC QLQ-C30 domains/symptom scales. The observed negative correlations between the WHOQOL-BREF and EORTC QLQ-C30 symptom scales indicate that a higher score of the WHOQOL-BREF domains corresponded with less worse symptoms.

**Table 4:** Correlations of the WHOQOL-BREF with the EORTC QLQ-C30 domains (*n* = 153)

EORTC QLQ-C30 domains/Items	WHOQOL-BREF domains/items				
	General facet	Physical Health	Psychological Health	Social Relationships	Environment
Physical Functioning	0.43*	0.73*	0.37*	0.04	0.26*
Role Functioning	0.46*	0.73*	0.46*	0.08	0.26*
Emotional Functioning	0.49*	0.51*	0.61*	0.19**	0.43*
Cognitive Functioning	0.33*	0.49*	0.47*	0.08	0.40*
Social Functioning	0.42*	0.59*	0.47*	0.07	0.32*
Global Health Status/QoL	0.67*	0.73*	0.58*	0.12	0.39*
Fatigue	− 0.39*	− 0.69*	− 0.44*	− 0.06	−0.33*
Nausea and vomiting	−0.32*	− 0.29*	− 0.24*	− 0.16	−0.05
Pain	−0.32*	− 0.62*	− 0.28*	− 0.06	−0.26*
Dyspnoea	− 0.28*	− 0.30*	− 0.15	0.05	−0.10
Insomnia	−0.29*	− 0.49*	− 0.35*	− 0.04	−0.32*
Appetite loss	− 0.36*	− 0.38*	− 0.22*	0.02	−0.05
Constipation	−0.17**	− 0.23*	− 0.18**	− 0.06	−0.24*
Diarrhea	0.00	−0.08	− 0.04	−0.10	− 0.06
Financial problems	−0.03	− 0.34*	− 0.22*	− 0.11	−0.36*

Pearson correlation coefficients

*P < 0.01

**P < 0.05

*Abbreviations: WHOQOL-BREF* World Health Organization Quality of Life-BREF questionnaire, *EORTC QLQ-C30* European Organization for Research and Treatment of Cancer Quality of Life Questionnaire Core 30, *n* number of patients, *QoL* quality of life

Table 5 shows the known-groups validity comparisons for the WHOQOL-BREF domains and General Facet in relation to the number of different adverse events, the number of different grade 3 or 4 adverse events, the ECOG performance status and the Global Health Status/QoL score. Significant differences were detected regarding the General Facet score, Physical Health, and Psychological Health among ECOG grades 0, 1, and 2 or higher. Similar results were observed for the General Facet and the WHOQOL-BREF domain scores according to Global Health Status/QoL as measured by the EORTC QLQ-C30 except for Social Relationships. For all of the observed significant differences except one (i.e. Psychological Health based on ECOG performance score), effect sizes were medium to large.

**Table 5:** Known-groups comparisons for the WHOQOL-BREF general facet and domain scores (*n* = 153)

	General Facet			Physical Health			Psychological Health			Social Relationships			Environment		
	*n*	Mean (SD)	*P*-value (effect size)*	*n*	Mean (SD)	*P*-value (effect size)*	*n*	Mean (SD)	*P*-value (effect size)*	*n*	Mean (SD)	*P*-value (effect size)*	*n*	Mean (SD)	*P*-value (effect size)*
Number of different adverse events^a^															
0–5	84	6.1 (1.7)	0.10	87	13.6 (3.1)	< 0.001 (0.08)	87	14.5 (2.5)	0.47	87	16.1 (2.7)	0.37	87	15.8 (2.2)	0.81
more than 5	66	5.6 (1.8)		66	11.8 (2.9)		66	14.2 (2.6)		66	16.4 (2.4)		66	15.9 (2.4)	
Number of different adverse events with CTCAE grade 3 or 4^a^															
0	112	6.0 (1.8)	0.16	115	13.1 (3.1)	0.06	115	14.4 (2.4)	0.93	115	16.1 (2.6)	0.36	115	15.8 (2.3)	0.82
1	27	6.0 (1.8)		27	12.8 (3.0)		27	14.4 (2.8)		27	16.5 (2.6) 17.1 (2.2)		27	16.0 (2.3)	
2 or more	11	4.9 (1.4)		11	10.8 (2.6)		11	14.7 (2.9)		11	16.3 (2.5)		11	16.1 (2.6)	
ECOG performance score^b^											16.5 (2.3)				
0	37	6.6 (1.5)	< 0.001 (0.08)	39	15.1 (2.3)	< 0.001 (0.23)	39	15.1 (2.2)	0.04 (0.04)	39	14.7 (3.5)	0.05	39	16.1 (1.8)	0.38
1	98	5.7 (1.8)		99	12.3 (3.0)		99	14.3 (2.6)		99			99	15.9 (2.5)	
2 or more	13	4.8 (1.1)		13	10.2 (2.0)		13	13.1 (2.2)		13			13	15.1 (2.3)	
Global health status/QoL EORTC QLQ-C30 score															
0–50	67	4.9 (1.5)	< 0.001 (0.30)	69	10.8 (2.7)	< 0.001 (0.36)	69	13.1 (2.6)	< 0.001 (0.22)	69	16.0 (2.8)	0.28	69	15.1 (2.4)	< 0.001 (0.10)
more than 50	79	6.8 (1.4)		80	14.6 (2.3)		80	15.5 (1.9)		80	16.5 (2.3)		80	16.6 (2.0)	

*Abbreviations: WHOQOL-BREF* World Health Organization Quality of Life-BREF questionnaire, *SD* standard deviation, *n* number of patients who completed the questionnaire, *CTCAE* Common Terminology Criteria for Adverse Events, *ECOG* Eastern Cooperative Oncology Group, *QoL* quality of life, *EORTC QLQ-C30* European Organisation for Research and Treatment of Cancer Quality of Life Questionnaire Core 30

*P*-values calculated with one-way ANOVA unless stated otherwise

*Effect sizes (η^2^) were only shown where one-way ANOVA was significant (*P* < 0.05).

^a^Reported adverse events: as reported at and before day 1 of the first cycle of chemotherapy

^b^Post hoc analyses with Tukey HSD test of significant differences: for the General Facet, between ECOG 0 and 1 and also 0 and 2 or higher; for Physical Health, between all ECOG categories; for Psychological Health, between ECOG 0 and ECOG 2 or higher

### Minimal clinically important differences

Table 6 demonstrates the distribution-based estimates of the MCIDs for the different domains of the WHOQOL-BREF.

**Table 6:** Estimates of minimal clinically important differences on WHOQOL-BREF domains

Domains	0.5 SD	1 SEM
General Facet	0.876	
Physical Health	1.545	2.155
Psychological Health	1.259	1.914
Social Relationships	1.274	2.716
Environment	1.142	1.761

*Abbreviations: WHOQOL-BREF* World Health Organization Quality of Life questionnaire, *SD* standard deviation, *SEM* standard error of measure

## Discussion

Patients with advanced disease lung cancer are prone to a decrease in QoL due to poor prognosis and cancer and treatment related adverse events. Unfortunately, trials investigating new therapies and treatment modalities in lung cancer often assess the impact on QoL with the use of HRQoL instruments. [2, 24–26]. This is unfortunate as the WHOQOL-BREF may facilitate a more comprehensive evaluation of QoL. Given the importance of a comprehensive evaluation of QoL, the present study assessed the psychometrics and MCIDs of the WHOQOL-BREF in patients with advanced lung cancer to facilitate adequate QoL monitoring in clinical practice and lung cancer trials. In general, our study demonstrated that the WHOQOL-BREF is a reliable and valid instrument in patients with advanced lung cancer.

We found that the General Health item of the General Facet was more positively skewed in our study compared with the WHOQOL-BREF field trial reflecting higher frequencies of patients with worse general health [5]. This is as expected given the frequent occurrence of adverse events and poor prognosis of advanced lung cancer. However, the patients in this study indicated better QoL for several items of the Psychological Health, Social Relationships and Environment domains than the patients included in the field trial. Moreover, an additional seven items of these three domains were negatively skewed in our patients indicating also better QoL. One item (i.e. Negative Feelings) was positively skewed demonstrating that most patients rarely experienced negative feelings while the WHOQOL-BREF field trial observed higher frequencies in the scale points that corresponded with increased negative feelings. As this higher level of QoL was not related to physical QoL, which is in general determined by universal factors (i.e. the cancer and its treatment), but rather to the other domains of the WHOQOL-BREF, this may be explained by several reasons. Given the negative skewness of seven of the eight items of the Environment domain, it is likely that the high standard of care and the high level of prosperity in the Netherlands may be, at least in part, responsible for this observation. In addition, patients with lung cancer may experience less psychological distress compared to patients with other types of cancer. A meta-analysis by Krebber et al. found that the prevalence of depression as diagnosed by a structural interview was the lowest (3%) in lung cancer patients compared with other forms of cancer. The prevalence of depression as diagnosed by self-report instruments (20%) was also lower or comparable with other forms of cancer [27].

Prior to testing the reliability and validity of the WHOQOL-BREF, we performed a first order CFA to analyse if the proposed four factor model matched with the patients in the present study. Before (i.e. RMSEA) and after (i.e. CFI and RMSEA) adding error covariances between the measurement errors of items Pain and Medication and between items Positive Feelings and Spirituality, the observed fit indices indicated a slightly better model fit than the field trial of the WHOQOL-BREF [5]. However, as we were not able to calculate 95% confidence intervals for the observed fit indices and Skevington et al. did not report them [5], we could not determine if the CFI and RMSEA observed in the present study were significantly different. Moreover, if they are different, it is likely that the differences in fit indices are explained by the differences between patient populations of both studies. In the present study a homogeneous sample of patients with advanced disease lung cancer was used whereas the patient population of the WHOQOL-BREF field trial consisted of patients with different diseases [5]. Also the statistical differences between the present study and that of Skevington et al. impair the direct comparison of model fit.

Similarly as observed by Skevington et al., the internal consistency of the Social Relationships domain was below the commonly accepted value of 0.7 [5] whereas the other domains all had a Cronbach’s alpha > 0.70. As Cronbach’s alpha is in part dependent of the number of items of a domain, a reason for this low alpha possibly lies in the fact that the Social Relationships domain consists of just three items. In a recent Taiwanese validation study of the WHOQOL-BREF which did not report specific results of patients with advanced disease lung cancer (i.e. overall results of Rasch analyses of patients with stage I to IV disease were reported), the inclusion of one extra item (i.e. Being Respected) in the Social Relationship domain resulted in a Cronbach’s alpha of 0.67 [12], which is higher than observed in this study, although comparable with the alpha found in the field trial (0.68) [5]. Explanations for the lower observed internal consistency of the Social Relationships domain in our study in contrast with the other two reports could be the homogeneity of the patient sample or the decreased ability of the combined items to reflect the underlying construct in patients with advanced disease lung cancer compared to those with limited disease stage or other forms of cancer or chronic diseases. Furthermore, one of the three items (i.e. Personal Relations) had a higher correlation with the Environment domain than with its own hypothesized domain in this study which indicates that this item may not be completely representative for the construct of Social Relationships. In addition to the relatively low Cronbach’s alpha, this result further hampers the interpretation of analyses with this domain and raises the question if the three items should be assessed separately.

After performing multi-trait/multi-item analyses we observed similar cross domain correlations as the field trial did. While the Self-esteem item of the Psychological Health domain in the field trial was strongly related with the other three domains [5], we observed a stronger correlation with the Physical Health domain than with its own domain. This is not only in contrast with the results of the field trial [5], but also with patients with other forms of cancers. One study in cervical cancer survivors reported that self-esteem was related to the mental component summary score and not with the physical component summary score of the Short Form 36 QoL questionnaire [28]. A reason for this result could be the considerable impact advanced lung cancer can have on physical abilities. This may lead to dependence of others which could affect self-esteem. In the field trial of the WHOQOL-BREF the centre specific analyses revealed that the items Safety & Security and Energy often had higher correlations with domains other than their own [5] whereas we found that this was the case for the items Sleep, Body Image, Self-esteem, and Personal Relations. These differences in cross-correlation could be explained by some reasons. For instance, as the sample size of this study was relatively small, the observed differences may reflect mere chance than a true observation. Also methodological differences and the specific characteristics of patients with advanced disease (e.g. poor prognosis, prone to cancer related adverse events) are, at least in part, responsible for these findings.

In general, low correlations were observed between WHOQOL-BREF domains and EORTC QLQ-C30 domains/ symptom scales. This is probably related to differences in constructs and concepts between the questionnaires. Whereas the WHOQOL-BREF is a generic questionnaire, the EORTC QLQ-C30 is a cancer specific questionnaire. Moreover, items of the WHOQOL-BREF are positively phrased while those of the EORTC QLQ-C30 are often negatively phrased. In this regard, the EORTC QLQ-C30 may not be regarded as a gold standard to evaluate construct validity of the WHOQOL-BREF. In addition, this also points to the additional value of the WHOQOL-BREF in QoL analyses in cancer patients.

Both the field trial of the WHOQOL-BREF and the recent Taiwanese study did not report MCIDs to facilitate the clinical application of the WHOQOL-BREF [5, 12]. In the present study, we were able to report statistically derived MCIDs for the four WHOQOL-BREF domains. Because we were not able to perform a test-retest reliability analysis, we used the conservative lower bound of the 95% confidence intervals of each of the Cronbach’s alphas of the WHOQOL-BREF domains for the calculation of the 1 SEM MCIDs. Considering the vulnerability of patients with advanced lung cancer for treatment and cancer related adverse events and the short period of 3 weeks between chemotherapy cycles, we expected it to be difficult to define an appropriated interval between completions of the WHOQOL-BREF for two reasons. 1) If the interval between completions of the WHOQOL-BREF would be too short, patients could recall their earlier answers. 2) If the interval between completions of the WHOQOL-BREF would be too long, it is likely that the occurrence of therapy and cancer related adverse events would have biased WHOQOL-BREF scores. However, by taking the lower bound of the confidence interval, we expected that patients who experience a larger difference over time than the observed SEM estimates are likely to have a true change. Considering that the 0.5 SD MICDs depend on the variance of test scores, which are expected to be relatively small in a homogenous patient population as in the present study, the larger 1 SEM MCIDs may provide a more conservative method for the interpretation of individual scores. However, 1 SEM MCIDs depend on the reliability of a questionnaire. A questionnaire with a limited reliability may result in a relatively large 1 SEM. This could result in an overestimation of the true MCID which decreases sensitivity. Given these considerations, we calculated MCIDs according to both methods and recommended to base the choice for either of the two approaches on the homogeneity of the patient sample and the reliability of the questionnaire in the particular population.

Another limitation is that the present study used CFA in combination with the multi-trait/multi-method methodology [14, 15] which is in contrast with the increased application of Rasch analysis in recent years to assess psychometric properties of QoL questionnaires in cancer [29–32]. However, we chose the same methodology for the analyses to enable precise comparisons of the psychometric properties observed in this study with those reported by the original field trial of the WHOQOL-BREF.

Lastly, the sample size of our study could be considered a limitation. Although we included less than recommended 200 patients by Boomsma and Hoogland [33], we still observed an acceptable model fit which demonstrated that our data suited the simple design of the model [34].

## Conclusions

This study demonstrated that the WHOQOL-BREF has satisfactory reliability and validity in patients diagnosed with advanced disease lung cancer. Moreover, we identified and proposed MCIDs to facilitate application of the WHOQOL-BREF not only in studies investigating new therapies and treatment modalities, but also in daily clinical practice.

## Abbreviations

ANOVA: Analysis of variance
CFA: Confirmatory factor analysis
CFI: Comparative Fit Index
CTCAE: Common Terminology Criteria for Adverse Events
ECOG: Eastern Cooperative Oncology Group
EORTC QLQ-C30: European Organisation for Research and Treatment of Cancer Quality of Life Questionnaire Core 30
HRQoL: Health Related Quality of Life
MCID: Minimal clinically important difference
QoL: Quality of Life
RMSEA: Root Mean Square Error Approximation
SD: Standard deviation
SEM: Standard error of measurement
WHOQOL-BREF: World Health Organization Quality of Life-BREF
